# Rickets guidance: part II—management

**DOI:** 10.1007/s00467-022-05505-5

**Published:** 2022-03-29

**Authors:** Dieter Haffner, Maren Leifheit-Nestler, Andrea Grund, Dirk Schnabel

**Affiliations:** 1 Department of Pediatric Kidney, Liver and Metabolic Diseases, Hannover Medical School, Carl-Neuberg-Str. 1, 30625 Hannover, Germany; 2 Pediatric Research Center, Hannover Medical School, Carl-Neuberg-Str. 1, 30625 Hannover, Germany; 3 Center for Chronically Sick Children, Pediatric Endocrinology, Charitè, University Medicine, Berlin, Germany

**Keywords:** Rickets, Management, Vitamin D, Phosphate, Nephrocalcinosis, Fanconi syndrome, Burosumab, Fibroblast growth factor 23, X-linked hypophosphatemia, Vitamin D-dependent rickets, Nutritional rickets

## Abstract

Here, we discuss the management of different forms of rickets, including new therapeutic approaches based on recent guidelines. Management includes close monitoring of growth, the degree of leg bowing, bone pain, serum phosphate, calcium, alkaline phosphatase as a surrogate marker of osteoblast activity and thus degree of rickets, parathyroid hormone, 25-hydroxyvitamin D_3_, and calciuria. An adequate calcium intake and normal 25-hydroxyvitamin D_3_ levels should be assured in all patients. Children with calcipenic rickets require the supplementation or pharmacological treatment with native or active vitamin D depending on the underlying pathophysiology. Treatment of phosphopenic rickets depends on the underlying pathophysiology. Fibroblast-growth factor 23 (FGF23)-associated hypophosphatemic rickets was historically treated with frequent doses of oral phosphate salts in combination with active vitamin D, whereas tumor-induced osteomalacia (TIO) should primarily undergo tumor resection, if possible. Burosumab, a fully humanized FGF23-antibody, was recently approved for treatment of X-linked hypophosphatemia (XLH) and TIO and shown to be superior for treatment of XLH compared to conventional treatment. Forms of hypophosphatemic rickets independent of FGF23 due to genetic defects of renal tubular phosphate reabsorption are treated with oral phosphate only, since they are associated with excessive 1,25-dihydroxyvitamin D production. Finally, forms of hypophosphatemic rickets caused by Fanconi syndrome, such as nephropathic cystinosis and Dent disease require disease-specific treatment in addition to phosphate supplements and active vitamin D. Adjustment of medication should be done with consideration of treatment-associated side effects, including diarrhea, gastrointestinal discomfort, hypercalciuria, secondary hyperparathyroidism, and development of nephrocalcinosis or nephrolithiasis.

## General approach

The primary treatment goal is to correct or at least improve rickets/osteomalacia based on clinical and biochemical parameters (Table 1) [1–4]. Clinical assessment includes growth parameters (height, weight, calculation of annual height velocity, and head circumference in infants), degree of leg bowing, gait pattern, presence of bone pain and muscle weakness, and dental abnormalities [5]. In addition, other disease-specific features, such as the presence of craniosynostosis and sensorineural hearing loss in children with X-linked hypophosphatemia (XLH) should be assessed (vide infra). Biochemical measures include serum phosphate, calcium, and alkaline phosphatase (ALP) as a surrogate marker of osteoblast activity and thus degree of rickets, parathyroid hormone (PTH), and 25-hydroxyvitamin D_3_ (25(OH)D). The latter is important in order to confirm adequate supplementation with native vitamin D in nutritional rickets and exclude vitamin D deficiency in other forms of rickets, which may hamper healing of rickets. In children with rickets, routine radiological assessments are not recommended and should be limited to situations where patients show insufficient clinical and/or biochemical response to medical treatment and if the results could lead to changes of management (e.g., drug dosages, orthopedic surgery). Suitable radiological methods are given in part 1 of this review [5].

**Table 1:** Suggested monitoring in patients with rickets requiring long-term treatment

Examination	0–5 years	5 years–start of puberty (9–12 years)	Puberty
Frequency of visits	Monthly–3 monthly	3–6 months	3 months
Height, weight, IMD, ICD^a^	✓	✓	✓
Head circumference and skull shape	✓	NA	NA
Presence of rickets, pain, stiffness, fatigue, muscle weakness, gait pattern	✓	✓	✓
Neurological examination^b^	✓	✓	✓
Orthopedic examination	Once a year in the presence of significant leg bowing		
Dental examination	Twice-yearly after tooth eruption	Twice-yearly	Twice-yearly
Hearing test^c^	Not feasible	From 8 years: hearing evaluation if symptoms of hearing difficulties	
Serum levels of ALP, Ca, Pi, PTH, Crea; eGFR	✓	✓	✓
25(OH) vitamin D levels	After 3–4 weeks in nutritional rickets, yearly in other rickets forms		
1,25(OH)_2_ vitamin D levels	Every 3–6 months in patients on burosumab treatment, those with HHRH or hypophosphatemia and nephrocalcinosis, and VDDR patients on active vitamin D		
U_Ca/Crea_^d^ TmP/GFR	Every 3–6 months in patients on active vitamin D or burosumab treatment. Initially, at every visit in patients on burosumab treatment		
Blood pressure	Twice yearly		
Kidney ultrasonography	Every 1–2 years on phosphate, active vitamin D or burosumab treatment		
Left wrist and/or lower limbs radiographs	- If leg bowing does not improve upon treatment	In adolescents with persistent lower limb deformities when they are transitioning to adult care	
	- If surgery is indicated		
Dental orthopantomogram	Not feasible	Based on clinical needs	
Funduscopy and brain MRI	If aberrant shape of skull, headaches or neurological symptoms	If recurrent headaches, declining school/cognitive performances or neurological symptoms	

IMD, intermalleolar distance; ICD, intercondylar distance; NA, not applicable; ALP, alkaline phosphatase; Ca, calcium; Pi, phosphorus; PTH, parathyroid hormone; Crea, creatinine; eGFR, estimated glomerular filtration rate[6]; U, urine; TmP/GFR, maximum rate of tubular reabsorption of phosphate per glomerular filtration rate; TmP/GFR is calculated by entering the fasting urine and plasma concentrations, in the same concentration units, into the following equation: TmP/GFR = Pp – (Up/ Ucr) × Pcr [7, 8]; an online calculator and reference values are available at: https://gpn.de/service/tmp-gfr-calculator/; HHRH, hereditary hypophosphatemic rickets with hypercalciuria; vitamin D-dependent rickets type (VDDR); table adapted from Haffner et al. [9]

^a^In the presence of significant leg bowing

^b^Consequences of craniosynostosis and spinal stenosis

^c^In patients with XLH and other inherited forms of fibroblast growth factor 23-associated hypophosphatemic rickets

^d^Upper normal range (mol/mol): 2.2 (< 1 year), 1.4 (1–3 years), 1.1 (3–5 years), 0.8 (5–7 years), 0.7 (> 7 years); normalization of initially low urinary calcium excretion approves adequate calcium intake

**Table 2:** Treatment doses of vitamin D_3_ or D_2_ for nutritional rickets

Age	Daily dose for 90 days IU	Single dose IU	Maintenance daily dose IU
< 3 months	2000	N/A	400
3–12 months	2000	50,000	400
> 12 months to 12 years	3000–6000	150,000	600
> 12 years	6000	300,000	600

Response to treatment should be assessed after 3 months since patients may require further treatment. A daily calcium intake of at least 500 mg should be ensured. For conversion from IU to μg, divide by 40. Table adapted from Munns et al. [10]

*N/A* not available

For physiological skeletal mineralization, infants aged 0–6 months (6–12 months) require a calcium intake of 200 mg/day (260 mg/day), and children and adolescents require 500 mg/day, respectively, and a 25(OH)D level greater than 50 nmol/L (20 ng/L) [10]. This must be assured in any child presenting with rickets. The cornerstone of treatment of calcipenic rickets is the supplementation or pharmacological treatment with native or active vitamin D depending on the underlying pathophysiology in combination with adequate calcium supplementation [10–13]. Treatment of phosphopenic rickets depends on the underlying pathophysiology. Fibroblast-growth factor 23 (FGF23)-associated hypophosphatemic rickets was historically treated with frequent doses of oral phosphate salts in combination with active vitamin D—so-called conventional treatment [9, 14, 15]. In addition, burosumab, a fully humanized FGF23 antibody, was recently approved for treatment of X-linked hypophosphatemia (XLH) and tumor-induced osteomalcia (TIO) [16, 17]. Other forms of FGF23-driven rickets currently continue to be treated with conventional therapy. Forms of hypophosphatemic rickets independent of FGF23, due to genetic defects of renal tubular phosphate reabsorption such as hypophosphatemic rickets with hypercalciuria (HHRH), are treated with phosphate only, as these diseases are associated with increased renal 1,25(OH)_2_D synthesis [18].

Normalization of serum ALP, calcium, phosphate, and PTH levels indicates healing of rickets. However, in severe forms of phosphopenic rickets, such as XLH, normalization of serum phosphate is not a practical goal during conventional treatment as serum phosphate levels quickly decrease again after each phosphate dose [9, 14, 15, 19, 20]. Such an approach would even be dangerous due to the increased risk of secondary hyperparathyroidism. Thus, in XLH patients on conventional treatment, improvement/normalization of serum ALP is the main biochemical surrogate indicating adequate treatment. By contrast, burosumab treatment is primarily tailored according to fasting serum phosphate levels, which should be kept in the lower age-related normal range. In addition, monitoring of renal phosphorus threshold concentration (TmP/GFR) is recommended in these patients, in order to confirm improvement of renal phosphate wasting [9]. Monitoring of FGF23 serum levels is not recommended in patients on burosumab treatment, as current assays cannot discriminate between burosumab-bound and free FGF23, resulting in unreliable results [21]. Normalization of initially low urinary calcium excretion, which is especially the case in children with calcipenic rickets, approves adequate calcium intake. In addition, patients should be monitored for treatment-associated side effects, e.g., hypercalciuria, nephrocalcinosis, nephrolithiasis, secondary hyperparathyroidism or suppressed PTH levels in patients receiving phosphate and/or vitamin D treatment, and hyperphosphatemia or hypervitaminosis D (1,25(OH)_2_D) in patients on burosumab treatment. Age- and sex-dependent normal values for routine biochemical parameters and important pitfalls in their assessment are provided in part 1 of this review [5]. Finally, regular monitoring for hypertension, which may be associated with the primary disease or treatment is recommended [9, 22].

## Calcipenic rickets

### Nutritional rickets

Patients should be treated with ergocalciferol (vitamin D_2_) or cholecalciferol (vitamin D_3_) at a minimal dose of 2000 IU (50 μg) per day in conjunction with 500 mg oral calcium per day, either as a dietary intake or supplements, for a minimum of 3 months (Table 2) [10, 23]. The latter allows adequate remineralization of the skeleton and prevents symptomatic hypocalcemia. Depending on the severity of rickets, vitamin D and calcium supplementation may already result in normalization of serum calcium and phosphate levels and a significant decrease in PTH levels within 3 weeks, whereas normalization of ALP levels may take several months [24]. The duration of therapy should be individually tailored, based on treatment response. Combined treatment is recommended, as the diet of children and adolescents with nutritional rickets is usually low in both vitamin D and calcium [25–28]. Oral vitamin D treatment is preferable, as it was shown to restore 25(OH)D levels more rapidly than intramuscular treatment, at least in adults [29]. In case of non-adherence vitamin D stoss therapy, e.g., single intramuscular injection ranging from 50,000 IU from the age of 3 months onwards up to 300,000 IU after the age of 12 years, may be used instead of oral vitamin D [10]. It must be stated that stoss therapy is associated with an increased risk of hypercalcemia [30]. Intravenous calcium gluconate should be given in patients with symptomatic hypocalcemia until normalization of serum calcium levels [31]. After stabilization of normocalcemia, the patient may be switched to oral calcium supplementation. Patients presenting with dilative cardiomyopathy are usually treated with diuretics and angiotensin-converting enzyme inhibitors and require management by a pediatric cardiologist [31, 32]. Finally, adequate nutritional requirement for vitamin D through diet and/or supplementation, which is at least 600 IU/day after the age of 12 months, should be assured after healing of rickets [10].

### Vitamin D-dependent rickets (VDDR)

#### Vitamin D-dependent rickets type 1A (VDDR1A)

In patients with vitamin D-dependent rickets type 1A, which is due to mutations in *CYP27B1*, the gene encoding 1-alpha hydroxylase, patients are treated lifelong with physiologic 1,25-dihydroxyvitamin D (1,25(OH)_2_D) doses, given twice daily due to its short half-life [13, 33]. Alternatively, alphacalcidiol (1alpha (OH)D) may be commenced which is converted in the liver to 1,25(OH)_2_D (calcitriol) and can be given once daily owing to its longer half-life. Due to the high calcium demands of the unmineralized skeleton, patients should be treated during the first 3 to 6 months with 2–5 times higher dosages of active vitamin D than needed during maintenance treatment (Table 3) [13, 34]. In addition, oral calcium supplementation (50 mg per kg body weight per day of elemental calcium) is recommended during the early phase of treatment to prevent aggravation of hypocalcemia due to bone remineralization (“hungry bones”). Later, normal dietary calcium intake should be ensured to allow optimal skeletal mineralization. Dosages of active vitamin D should be tailored to keep serum PTH and calcium levels in the mid-normal range.

#### Vitamin D-dependent rickets type 1B (VDDR1B)

Patients with vitamin D-dependent rickets type 1B, which is due to mutations in *CYP2R1* resulting in impaired 25-hydroxylation of vitamin D_2_ and vitamin D_3_ to 25(OH)D should be treated with calcidiol (also called calcifediol or 25-hydroxy-vitamin-D), which bypasses the defect in 25-hydroxylation, plus supplemental calcium [1, 13, 35]. The latter should be done as for VDDR1A patients. Alternatively, pharmacological dosages of vitamin D_2_ or vitamin D_3_ or physiological doses of calcitriol can be given, again plus calcium supplementation. The advantage of calcifediol appears to be that it restores diminished serum concentrations of 25(OH)D allowing physiological control of its conversion to calcitriol by PTH secretion. Drug doses should be tailored to maintain serum levels of circulating calcium and PTH in the mid-normal range. Physicians need to appreciate that the phenotype is milder in patients with one defective allele and that it usually improves with age [36].

#### Vitamin D-dependent rickets type 2A (VDDR2A)

Vitamin D-dependent rickets type 2A (VDDR2A) is due to mutations in *VDR* resulting in impaired signaling of the vitamin D receptor. Intestinal absorption of calcium is largely independent of vitamin D during early infancy [37]. Therefore, high oral doses of calcium (5–6 g/m^2^ body surface area of elemental calcium) are usually sufficient to restore normocalcemia and normalize PTH levels during the first few months of life in infants with VDDR2A [13]. However, some patients require primary intravenous calcium infusions to adequately raise serum calcium. Calcium infusions need to be continued until the “hungry bone syndrome” is cured, i.e., the point at which oral calcium supplementation allows normocalcemia to be maintained. After the early infancy period, patients require additional treatment with vitamin D, preferentially with calcitriol or alphacalcidiol, alternatively with vitamin D_3_, vitamin D_2_, or 25(OH)D_3_ which act as substrates for calcitriol generation [38]. However, the response is highly variable. As a rule of thumb, children with normal hair have milder forms than those with alopecia and may show complete remission when receiving vitamin D at doses given in Table 3 [13, 38]. By contrast, about half of patients presenting with alopecia will be resistant even to the highest vitamin D doses. The other half will achieve normocalcemia but require 10 times higher vitamin D doses compared to those with normal hair. Accordingly, maintenance therapy is also very variable. Patients only responding to large amounts of active vitamin D benefit from additional supplementation with oral calcium (1000 mg of elemental calcium per day). Those patients not responding to high doses of active vitamin D require high doses of calcium, which usually cannot be provided by oral supplementation due to the limited tolerance to oral calcium (about 6 g per day in children), and diminished vitamin D-dependent calcium absorption in these patients [13]. Therefore, these patients may require intravenous calcium infusions (1000 mg of elemental calcium per day, given over 12 h) over many months until oral supplementation with calcium salts in conjunction with active vitamin D, allows for maintenance of both normocalcemia and adequate skeletal mineralization. During puberty, intestinal calcium absorption often improves in VDDR2A patients for so far unknown reasons, establishing stable normocalcemia when using moderate doses of oral calcium. During long-term follow-up, bone mineral density was shown to be normal and PTH levels could be kept close to the upper-normal range in the majority of VDDR2A patients [39, 40].

#### Vitamin D-dependent rickets type 2B (VDDR2B)

This disease is due to mutations in *HNRNPC* also resulting in impaired signaling of the vitamin D receptor. Therefore, treatment of VDDR2B patients should be similar to that of VDDR2A patients (vide supra) [1, 13, 41].

#### Vitamin D-dependent rickets type 3 (VDDR3)

This ultra-rare condition is due to mutations in *CYP3A4* leading to enhanced inactivation of calcitriol and was shown to respond to high doses of vitamin D_3_ or D_2_ as given in (Table 3). This allows normocalcemia and normalization of serum phosphate, PTH, and ALP serum levels in these patients [13, 42].

**Table 3:** Suggested vitamin D dose for maintenance treatment of patients with VDDR

	VDDR1A	VDDR1B	VDDR2	VDDR3
	(μg per day)	(μg per day)	(μg per day)	(μg per day)
Vitamin D_3_ or D_2_	NI	100–200	125–1,000?^a^	**1000 to?**
Calcidiol	NI	**20–50**	20–200^a^	50 to?
Calcitriol	**0.3–2**	0.3–2	**5–60**^b^	1 to?
Alphacalcidiol	**0.5–3**	0.5–3	**5–60**^b^	2 to?

Dose requirements are usually not dependent on body weight. Therefore, absolute doses are given. In addition, calcium supplementation is advised in all patients as outlined in the text. The preferred form of vitamin D is given in bold for each type of VDRR.

*NI* not indicated

^a^Patients with milder phenotypes (usually with normal hair) often can respond to analogs requiring 1-hydroxylation. Maximal useful doses are unknown. Serum 1,25(OH)_2_D should be maintained in the range of 200–1000 pg/mL

^b^Maximal doses are limited by cost and adherence; some patients do not respond despite maximal doses. Table adapted from Levine [13].

## Phosphopenic rickets

### Dietary phosphate deficiency or impaired availability

Adequate dietary or additional oral phosphate supplementation will correct dietary phosphate deficiency, whereas in case of impaired dietary phosphate availability, such as in infants on amino acid-based elemental formulas (e.g., Neocate®), a change of formula will cure rickets [43]. Management of impaired phosphate absorption, e.g., due to gastrointestinal surgery or short bowel syndrome, are treated with oral or if necessary parenteral phosphate supplementation (Tables 4 and 5).

**Table 4:** Daily doses for phosphate and active vitamin D (conventional treatment) in children with X-linked hypophosphatemia (XLH) and tumor-induced hypophosphatemia (TIO)

Drug	XLH	TIO
Phosphate^a^ (mg/kg)/(mmol/kg) given in 4–6 doses	20–60/0.7–2.0 Maximum 80 mg/kg	15–60/0.5–2
Calcitriol^b^ (ng/kg) given in 1–2 doses	20–30 Alternatively, 0.5 μg^c^ (age > 12 months)	15–60
Alphacalcidiol^b^ (ng/kg) given once daily	30–50 Alternatively, 1 μg^c^ (age > 12 months)	15–60

^a^Based on elemental phosphorus; infants and young children usually require more frequent phosphate administrations than older children and adolescents

^b^Phosphate should always be given in combination with either calcitriol or alphacalcidiol

^c^Starting dose; other forms of fibroblast-growth factor 23-associated hypophosphatemic rickets are usually treated with similar doses, but evidence-based recommendations or consensus statements are lacking; further details are given in the text; table adapted from Haffner et al. [9] and Florenzano [44]

**Table 5:** Burosumab treatment in children with X-linked hypophosphatemia (XLH) and tumor-induced hypophosphatemia (TIO)

	Treatment administration
Starting dose titration	• XLH: 0.8 mg/kg^a^ every 2 weeks subcutaneously
	• TIO: 0.8 mg/kg every 2 weeks subcutaneously
	• 0.4 mg/kg increments to raise fasting serum phosphate levels to within the lower end of the normal reference range for age, to a maximum dosage of 2.0 mg/kg body weight (maximal dose: XLH, 90 mg; TIO, 180 mg))
	• Burosumab should not be adjusted more frequently than every 4 weeks
	• Burosumab dosage should be switched to the regimen recommended for adult XLH patients, i.e., 1 mg/kg (maximum dose 90 mg) given every 4 weeks subcutaneously, after end of skeletal growth (height velocity < 1 cm/year or epiphyseal closure on X-ray)
Monitoring of fasting serum phosphate^b^	• XLH: Every 2 weeks during the first month, every 4 weeks during the following 2 months and thereafter as appropriate:
	– During titration period: ideally, 7–11 days after the last injection to detect hyperphosphatemia
	– After achievement of a steady-state (which can be assumed after 3 months of a stable dose) preferentially, directly before injections to detect underdosing
	• TIO: Initially every 4 weeks, ideally 2 weeks post last injection
Other dose recommendations	• Withhold dose if fasting serum phosphate level is above the upper range of normal
	• Burosumab may be restarted at approximately half of the previous dose when serum phosphate concentration is below the normal range
Contraindications	Do not administer:
	• Alongside conventional treatment
	• When fasting phosphate levels are within the age-related normal
	reference range before treatment initiation
	• In the presence of severe renal impairment
	• In sexually active adolescent females without adequate contraception

^a^The starting dose as originally approved by EMA amounted to 0.4 mg/kg and was later on increased to 0.8 mg/kg which is in line with current FDA approval. Burosumab is so far approved for treatment of TIO in children by the FDA and in Japan only

^b^Recommendations for general patient monitoring are given in Table 1. Further details are given in the text. Table adapted from Haffner et al. [9] and the Crysvita US prescribing information 2021 [45]

### FGF23-mediated renal phosphate wasting

#### X-linked hypophosphatemia (XLH)

X-linked hypophosphatemia is due to mutations in *PHEX* resulting in increased expression of FGF23 in bone and consecutive renal phosphate wasting and reduced calcitriol levels, as well as other so far poorly understood alterations [46]. Patients with XLH can either be treated with oral supplements of inorganic phosphate salts in combination with active vitamin D (“conventional treatment”) or with burosumab [9, 14, 47]. Burosumab has several advantages over conventional treatment—(i) it removes the burden of medicating many times a day, which markedly hampers adherence during conventional treatment; (ii) it was shown to be more effective in healing rickets; and (iii) it has a very good profile—whereas conventional treatment is associated with side-effects such as gastrointestinal discomfort, hypercalciuria, secondary hyperparathyroidism, diarrhea, and nephrocalcinosis [48–51]. However, burosumab is an expensive drug which hampers its availability. In addition, some patients with XLH may require rather low doses of phosphate salts and active vitamin D and may therefore not be mandatory candidates for burosumab treatment [16]. Until now, burosumab is not licensed for treatment in patients aged below 12 months (6 months in the USA), whereas older patients can already be treated initially with burosumab. Detailed clinical practice recommendations for the treatment of XLH were published recently and are briefly outlined below [9].

##### Conventional treatment

The starting dose of phosphate amounts to 20–60 mg/kg/day (0.7–2.0 mmol/kg per day) based on elemental phosphorus, given at least four times a day. Dosages should be adjusted according to clinical and biochemical responses [9]. High doses (> 80 mg/kg per day) are associated with gastrointestinal side effects (diarrhea, abdominal discomfort) and secondary hyperparathyroidism. Treatment with phosphate should always be done in combination with active vitamin D (either with calcitriol or alphacalcidiol) in XLH patients, as this prevents the development of secondary hyperparathyroidism as seen in patients treated with phosphate salts alone, which further promotes renal phosphate wasting and can result in autonomous (tertiary) hyperparathyroidism. Treatment with active vitamin D also improves remineralization of the skeleton by enhancing vitamin D-dependent intestinal calcium and phosphate absorption. The starting doses of calcitriol and alphacalcidiol amount to 20–30 ng/kg body weight and 30–50 ng/kg body weight daily, respectively. Alternatively, calcitriol and alphacalcidiol may be empirically started at 0.5 μg and 1 μg per day in patients aged above 12 months. Dosages need to be adjusted in order to keep PTH levels in the normal range and to avoid hypercalciuria. Increased PTH levels can either be managed by increasing the dosage of active vitamin D or by decreasing phosphate dosage. In case of hypercalciuria the dosages of active vitamin D should be reduced. Conventional treatment should be maintained until adult height is achieved. Patients showing inadequate response to conventional treatment or significant side-effects are potential candidates for burosumab treatment (vide infra). Supplementation with vitamin D_2_ or D_3_ is recommended in patients showing concomitant vitamin D deficiency as in the general population [9]. Finally, attention should also be paid to an age-appropriate daily calcium intake, i.e., at least 500 mg calcium in patients aged above 12 months as in other forms of rickets [10].

##### Burosumab treatment

Burosumab has been approved by the European Medicines Agency (EMA), the US Food and Drug Administration (FDA), and Japanese health authorities for treatment of pediatric XLH patients aged above 12 months (> 6 months in the USA), showing radiographic evidence of bone disease and with growing skeletons. A European XLH guideline recommends the initiation of burosumab treatment under the following conditions: “radiographic evidence of overt bone disease; disease that is refractory to conventional therapy; complications related to conventional therapy; or patient’s inability to adhere to conventional therapy, presuming that adequate monitoring is feasible” [9]. However, XLH patients may also be primarily started on burosumab if they meet the approval criteria of the local authorities. Important note: treatment with phosphate salts and active vitamin D must be discontinued at least one week before initiation of burosumab therapy to confirm hypophosphatemia. Finally, sexually active adolescent females should only receive burosumab if they are using adequate contraception.

The recommended starting dose of burosumab in children amounts to 0.8 mg/kg body weight. It should be given in 2-weekly intervals as subcutaneous injections. Burosumab should be titrated in 0.4 mg/kg increments to raise fasting serum phosphate levels into the lower end of the normal reference range for age with a maximal dose of 2.0 mg/kg body weight (maximum dose 90 mg). A steady state can be assumed after applying a constant dose for at least three months. Therefore, doses should only be increased after at least 4 weeks. We suggest monitoring serum fasting phosphate levels during the titration period between injections, ideally 7 to 11 days post injection, in order to detect hyperphosphatemia. Thereafter, serum phosphate levels should ideally be monitored directly before the next injection to exclude hypophosphatemia. In elevated serum phosphate levels, burosumab should be discontinued, but may be reinstated after normalization of serum phosphate levels using half of the previous dose [9]. Finally, the burosumab dosage should be switched to the regimen recommended for adult XLH patients, i.e., 1 mg/kg (maximum dose 90 mg) given every 4 weeks subcutaneously, after end of skeletal growth, i.e., at near final height (height velocity < 1 cm/year) and/or in case of epiphyseal closure on x-ray of the left wrist.

##### Adjunctive therapies

About half of all XLH patients show persistent short stature despite adequate conventional or burosumab treatment [9, 49, 52]. Several clinical studies have shown that treatment with recombinant human growth hormone (rhGH) increases growth rates and standardized height in short children with XLH [38–40]. However, in a small randomized clinical trial, near-final height did not significantly differ between rhGH-treated XLH patients and controls [53]. Therefore, routine treatment with rhGH in short children with XLH is not recommended [9].

##### Surgical management

About half of all XLH patients present with significant leg bowing despite conventional treatment and require surgical corrections [9, 54]. So far, no long-term data on the need for surgical corrections in XLH patients treated with burosumab are available. Children with persistent leg deformities should undergo thorough assessment by an experienced pediatric orthopedic surgeon on the need and optimal timing for surgical treatment, i.e., corrective osteotomies versus epiphysiodesis [9, 54–56]. The latter technique requires remnant growth potential and should be performed at least 2–3 years before the end of skeletal growth. By contrast, it was recommended that osteotomies should be carried out later in childhood or at attainment of adult height, in order to reduce complications, e.g., need for renewed surgery. In general, an optimal metabolic control should be assured before performing a surgical procedure in order to avoid recurrence of leg deformities.

##### Dental care

Pediatric XLH patients are prone to developing spontaneous dental abscesses causing pain and swelling in deciduous as well as in permanent teeth, due to poorly mineralized dentin [9, 57]. In addition, adolescent and adult XLH patients may develop significant periodontitis which may cause tooth loss. Treatment with oral phosphate and active vitamin D was shown to improve dentin mineralization, and to reduce the frequency of complications, i.e., dental abscesses and periodontitis [58, 59]. So far, the effects of burosumab on dental health in XLH patients is unknown. Children with XLH should undergo at least 6-monthly dental visits, including investigation for pulp necrosis (changes in color, presence of fistula, swelling, abscess, cellulitis, or pain) and, if indicated, additional radiological assessments. Patients may require sealing of pits and fissures with flowable resin. It is important to optimize treatment with oral phosphate and active vitamin D or burosumab first, in patients requiring orthodontic treatment [9].

##### Neurosurgical complications

Patients presenting with symptoms suggesting craniosynostosis, Chiari type 1 malformation and/or intracranial hypertension, e.g., dolichocephalus, persistent headache, occipital or neck pain, should undergo further investigation, including funduscopy and cranial imaging in consultation with a neurosurgeon [9, 60].

#### Autosomal-dominant hypophosphatemic rickets (ADHR)

This condition is due to mutations in *FGF23* resulting in impaired FG23 degradation and consequently FGF23-driven renal phosphate wasting. Patients with ADHR are usually treated with oral phosphate salts and active vitamin D, as with XLH patients [61]. However, the required dosages vary largely and need to be adapted according to clinical and biochemical response (vide supra). Iron deficiency was shown to promote the severity of the phenotype in XLHR patients. Iron repletion was shown to normalize previously elevated FGF23 levels and to improve serum phosphate levels in XLH patients [62]. Therefore, assessment and correction of any iron deficiency in ADHR patients is recommended. In addition, other potential complications such as dental abscesses need to be addressed as well (vide supra).

#### Autosomal-recessive hypophosphatemic rickets type 1 and 2 (ARHR 1 and 2)

Treatment with phosphate supplementation and active vitamin D was shown to improve rickets and normalize serum ALP levels in patients with ARHR1 which is due to mutations in the dentin matrix protein 1 (*DMP1*) gene [63]. However, the number of published cases is low. Treatment of patients with ARHR2 is challenging due to its rarity and the fact that ENPP1 deficiency not only causes hypophosphatemic rickets but may also be associated with arterial, cardiac and/or articular calcification or may present as generalized arterial calcification in infancy [64–67]. The latter phenotypes preclude treatment with active vitamin D. The development of calcification is due to the fact that proper ENPP1 function is required to generate pyrophosphate which is a major inhibitor of mineralization. Therefore, ARHR2 patients should undergo careful screening and monitoring for arterial/cardiac calcification including assessment of carotid intima media thickness and cardiac ultrasound. The presence of calcifications precludes treatment with active vitamin D. In a small series including 6 ARHR2 patients, the reported dosages of phosphate salts (based on elemental phosphate) and calcitriol amounted to 40 mg/kg day and 15 ng/kg per day, respectively [65]. In addition, patients with biallelic *ENPP1* mutations may develop normocalcemic primary hyperparathyroidism which may require partial parathyroidectomy [64].

Recent preclinical studies show that ENPP1 enzyme replacement therapy normalizes serum pyrophosphate levels and consequently prevents the development of complications due to *ENPP1* mutations in rodent models of generalized arterial calcification of infancy [68, 69]. Clinical trials are currently underway to assess the efficacy and safety of ENPP1 enzyme replacement therapy in patients with *ENPP1* mutations including ARHR2. The initially described effect of bisphosphonate therapy in GACI patients to reduce the early mortality risk could not be confirmed in the recently published study with historical data from 247 patients [70].

#### Raine syndrome (ARHR3)

Patients with Raine syndrome (ARHR3) which is due to *FAM20c* mutations rarely survive infancy [71]. Although, non-lethal cases of Raine syndrome have been described, these patients rarely presented with persistent rickets but rather with osteosclerosis of the long bones [71, 72]. Preclinical studies showed that a high-dose phosphate diet is able to improve bone mineralization in *Fam20c*-deficient mice [73]. Whether this is also effective in patients with non-lethal Raine syndrome showing persistent rickets needs to be proven.

#### Fibrous dysplasia (FD)

Treatment of patients with fibrous dysplasia (FD), which is due to activating mutations in the *GNAS* gene causing McCune–Albright syndrome primarily focuses on non-rickets complications [74]. Although small retrospective case series suggested the beneficial effects of antiresorptive treatment with bisphosphonates in patients with FD, a randomized clinical trial failed to show any significant improvement in clinically relevant outcome measures, including bone pain and fractures [75–77]. Therefore, this approach cannot generally be recommended [74]. Treatment with phosphate salts and active vitamin D may be considered in patients presenting with hypophosphatemic rickets due to renal phosphate wasting [74]. Burosumab treatment resulted in normalization of serum phosphate and ALP levels and improvement of clinical symptoms (bone pain, muscle weakness, walking ability) in a patient with FD showing persistent hypophosphatemia and skeletal complications despite oral supplementation with phosphate and treatment with active vitamin D [78]. Further studies must be awaited to prove the safety and efficacy of this measure in these patients.

#### Tumor-induced osteomalacia (TIO)

Complete tumor resection is the treatment of choice in pediatric and adult TIO patients [44, 79–82]. Serum FGF23 and phosphate levels were shown to normalize after tumor removal within 1 h and 5 days, respectively, but complete bone healing may take up to 12 months [83]. FGF23 monitoring can also be used to detect tumor recurrence, which may require a second excision. If there is no evidence of a local recurrence, patients should be evaluated by high-resolution CT scans for metastasis. Until surgery, patients should be started on treatment with phosphate salts and active vitamin D, which was shown to improve skeletal mineralization and ameliorate clinical symptoms. This treatment is also indicated in cases where the FGF23 producing tumor cannot be located, complete tumor removal is not possible or in patients with severe comorbidities. The recommend dose of phosphate salts amount to 15–60 mg/kg per day based on elemental phosphate, given in 4 to 6 doses [44]. Calcitriol or alphacalcidiol should be given at dosages of 15–60 ng/kg per day. Dosages should be adjusted according to the clinical and biochemical response and development of side effects as outlined above. Important to note; PTH levels may already be elevated at initial presentation which further promotes renal phosphate wasting and thus requires high doses of active vitamin D which can usually be reduced later.

As an alternative to conventional treatment, patients can also be treated very effectively with burosumab which was recently approved for treatment of TIO in children and adults by the FDA and the Japanese health authorities based on clinical trials in adult patients [17, 84]. As burosumab treatment, as with conventional treatment, does not stop tumor growth and thus the progression of diseases, it should be limited to cases where complete tumor removal is not possible or feasible. The recommended starting dose for treatment of TIO in children aged 2–18 years is 0.4 mg/kg of body weight by subcutaneous injections every 2 weeks [45]. Serum phosphate levels should initially be monitored every 4 weeks, ideally 2 weeks post last injection, to confirm that serum concentrations have risen to the age-related normal range and to detect hyperphosphatemia. Dosages may gradually be increased up to 2 mg/kg (maximum 180 mg) in cases of persistent low phosphate levels as in children with XLH. Finally, a recent case report suggests that targeted blockade of the FGF receptor (FGFR) may be a future suitable treatment option for TIO [85].

### Hypophosphatemic disorders with normal or suppressed FGF23 activity

#### Hereditary hypophosphatemic rickets with hypercalciuria (HHRH)

This condition is due to mutations in *SLC34A3* resulting in loss of function of NaPi2c in the proximal tubule and consequent renal phosphate wasting. Patients with HHRH are treated with renal phosphate salts only. Treatment with active vitamin D is not indicated in view of the already elevated 1,25(OH)_2_D levels [86, 87]. Primary target parameters on high phosphate supplementation are to normalize serum phosphate and 1,25 (OH)_2_D concentrations [16, 72]. Observational studies showed that treatment with phosphate supplements normalizes ALP and radiological surrogates of rickets in HHRH patients [86, 88]. However, it is unknown if long-term oral phosphate treatment is safe with respect to the development of nephrocalcinosis in HHRH patients, as high phosphate dosages were shown to be associated with increased risk of nephrocalcinosis in XLH patients for, so far, poorly understood reasons [9, 89, 90]. Therefore, careful monitoring is advised including serum phosphate, calcium, ALP, PTH, 1,25 (OH)_2_D, and urinary calcium excretion at 3 to 6 monthly intervals and yearly kidney ultrasound investigations. However, the safety and efficacy of long-term phosphate treatment needs to be proven. In addition, adjunctive measures like those in other hypercalciuria-associated forms of nephrolithiasis/nephrocalcinosis should be considered [91]. This includes a high fluid intake and the avoidance of a high dietary sodium and protein intake. Finally, thiazide treatment may be initiated to ameliorate hypercalciuria [92].

#### Hypophosphatemia and nephrocalcinosis

This disease is also due to a defect in a sodium-dependent phosphate transporter located in the proximal tubules (in this case NaPi2a encoded by *SLC34A1*) resulting in hypophosphatemia, suppressed FGF23 levels and excessive 1,25(OH)_2_D levels. Therefore, treatment should be as described for HHRH patients. A retrospective observational study of phosphate supplementation in 6 of 16 children with biallelic *SLC34A1* mutations showed rapid normalization of serum phosphate levels and parameters of calcium/vitamin D metabolism which was associated with improved weight gain [93].

#### X-linked recessive hypophosphatemic rickets: Dent disease 1

Dent disease also called X-linked recessive hypophosphatemic rickets is due to mutations in *CLCN5* resulting in loss of function of CLCN5 in the proximal tubule which is associated with renal phosphate wasting [94, 95]. Treatment of this condition primarily focuses on decreasing hypercalciuria and preventing nephrolithiasis, nephrocalcinosis and progressive chronic kidney disease, as described elsewhere [96]. The severity of hypocalcemia varies greatly in Dent patients. Treatment with phosphate supplements should be restricted to those patients presenting with clinical, biochemical and radiological signs of hypophosphatemic rickets and should aim to normalize serum ALP levels [94, 95].

##### Nephropathic cystinosis

A comprehensive international guideline on the management of bone disease in children with nephropathic cystinosis was published recently [97]. Nephropathic cystinosis is a lysosomal storage disease due to mutations in *CTNS* resulting in cystine accumulation in the proximal tubule and consecutive Fanconi syndrome and progressive chronic kidney disease which can be ameliorated by cysteamine therapy. Treatment of hypophosphatemic rickets primarily focuses on replacement of urinary losses due to Fanconi syndrome including water, bicarbonate, and phosphate which varies greatly. Patients require adequate caloric and protein intake, supplementation with native vitamin D for vitamin D deficiency, calcium supplementation in cases of persistent hypocalcemia and treatment with active vitamin D to help cure rickets. Patients may require physical therapy and orthopedic surgery for persistent significant limb deformities. Finally, treatment with rhGH should be considered in case of persistent short stature [98].

#### Iatrogenic proximal tubulopathy

The primary goal in children with hypophosphatemic rickets due to iatrogenic proximal tubulopathy should be to stop the injurious agent [99–102]. In addition, phosphate supplementation in combination with cautious treatment with active vitamin D for prevention of secondary hyperparathyroidism should be considered to heal rickets.

##### Key summary points

- An adequate calcium intake and normal 25-hydroxyvitamin D_3_ serum levels should be assured in all children with rickets.
- Children with calcipenic rickets require the supplementation or pharmacological treatment with native or active vitamin D depending on the underlying pathophysiology.
- Children with X-linked hypophosphatemia should be treated with burosumab, if available, or with frequent doses of oral phosphate salts in combination with active vitamin D as used for other forms of fibroblast-growth factor 23 (FGF23)-associated hypophosphatemic rickets.
- Patients with tumor-induced osteomalacia should primarily undergo tumor resection, if possible.
- Forms of hypophosphatemic rickets independent of FGF23 due to selective genetic defects of renal tubular phosphate reabsorption, are treated with oral phosphate only, since they are associated with excessive 1,25-dihydroxyvitamin D production.
- Adjustment of medication should be done with consideration of treatment-associated side effects, including diarrhea, gastrointestinal discomfort, hypercalciuria, secondary hyperparathyroidism, and development of nephrocalcinosis or nephrolithiasis.

##### Multiple choice questions

1. Which of the following statements is true?

- The cause of nutritional rickets is always a deficiency of vitamin D.
- Nutritional rickets can be excluded if the 1,25(OH)2D is highly normal or even outside the upper normal range.
- Therapy of nutritional rickets is exclusively done with high vitamin D supplementation.
- During monitoring of nutritional rickets, the drop in PTH is the earliest and most important indicator of response to therapy.
- In case of tetany due to nutritional rickets, rapid initiation of vitamin D supplementation is sufficient.

2) Which of the following statements is true?

- The marked hypophosphatemia which may be present in nutritional rickets requires oral supplementation with phosphate salts.
- The diagnosis of vitamin D-dependent rickets type 1B should be considered if high-dose therapy with native vitamin D does not lead to a normalization of 25-OHD and a concomitant decrease in PTH levels.
- Intramuscular injection of vitamin D is the treatment of choice in nutritional rickets.
- Vitamin D-dependent rickets type 2a and 2b differ with respect to response to calcitriol therapy.
- Administration of intravenous calcium is the therapy of choice in vitamin D-dependent rickets type 1B in the first months of life.

3) Which of the following statements is true?

- Monitoring of children with hypophosphatemic rickets treated with phosphate salts and active vitamin D includes the assessment of serum phosphate, calcium, alkaline phosphatase, creatinine, 1,25(OH)_2_ vitamin D, and calculation of maximum rate of tubular reabsorption of phosphate per glomerular filtration rate (TmP/GFR).
- Children with hypophosphatemic rickets should undergo yearly X-ray examinations of the left wrist and/or lower limbs.
- Patients with hypophosphatemic rickets due to defects in sodium-dependent tubular phosphate transporters should undergo yearly hearing tests.
- Calculation of maximum rate of tubular reabsorption of phosphate per glomerular filtration rate (TmP/GFR) is recommended to assess renal phosphate wasting in children on burosumab treatment.
- Assessment of PTH levels is not helpful in the management of children with hypophosphatemic rickets treated with burosumab.

4) Which of the following statements is true?

- Children with hypophosphatemic rickets are usually treated with phosphate salts and active vitamin D.
- Burosumab is the treatment of choice in infants aged less than 6 months with X-linked hypophosphatemia.
- Patients with hypophosphatemic rickets due to defects in sodium-dependent tubular phosphate transporters require no treatment with active vitamin D.
- Conventional treatment with phosphate salts and active vitamin D is more effective than burosumab treatment in patients with X-linked hypophosphatemia.
- Phosphate should be given in two divided doses in patients with X-linked hypophosphatemia.

5) Which of the following statements is true?

- High doses of phosphate and/or active vitamin D are associated with the development of nephrocalcinosis in children with hypophosphatemic rickets.
- The starting dose of burosumab in children with X-linked hypophosphatemia amounts to 0.4 mg/kg given every two weeks subcutaneously.
- The starting dose of burosumab in children with tumor-induced osteomalacia amounts to 0.8 mg/kg given every two weeks subcutaneously.
- Active vitamin D may be combined with burosumab for treatment of X-linked hypophosphatemia.
- Burosumab should be tailored in 0.4 mg/kg increments every two weeks to raise fasting serum phosphate levels to within the lower end of the normal reference range.
